# Insights into Neonatal Oral Feeding through the Salivary Transcriptome

**DOI:** 10.1155/2012/195153

**Published:** 2012-07-10

**Authors:** Jill L. Maron

**Affiliations:** Division of Newborn Medicine, Mother Infant Research Institute at Tufts Medical Center, Floating Hospital for Children at Tufts Medical Center, 800 Washington Street, P.O. Box 394, Boston, MA 02111, USA

## Abstract

*Background*. The development of safe and effective oral feeding skills in the newborn is complex and may be associated with significant morbidities. Our understanding of neonatal oral feeding maturation at the molecular level is limited, providing an opportunity to utilize emerging molecular techniques to accurately assess neonatal oral feeding skills. *Objective*. To identify key regulatory genes in neonatal saliva involved in successful oral feeding. *Methods*. Previously, our laboratory identified 9,286 genes in saliva that statistically significantly altered their gene expression as premature newborns gained advanced oral feeding skills. In this report, genes previously identified underwent an updated and targeted pathway analysis with Ingenuity Pathway Analysis (IPA) to identify potential candidate genes involved in successful oral feeding. Genes were considered if they were in the five most significantly up- and down-regulated physiological pathways and were associated with the keywords “feeding”, “digestion” and “development”. *Results*. There were 2,186 genes that met criteria. Pathways associated with feeding behavior, cranial nerve development, and the development of the nervous, skeletal, and muscular systems were highlighted. *Discussion*. These data provide important insights into the biological processes involved in oral feeding in the newborn at a molecular level and identify novel pathways associated with successful oral feeding.

## 1. Introduction

The vast majority of infants admitted to the neonatal intensive care unit (NICU) must acquire the skills for successful oral feeding prior to discharge. For the preterm infant, this complex task is not without risks. Successful oral feeding involves the maturation and integration of the nervous, sensory, muscular, and digestive systems. Failed oral feeding trials can result in an array of morbidities including choking, aspiration, bradycardia, desaturations, feeding aversions, and both short- and long-term impaired neurological outcomes [1–4]. Further, there is a subset of term infants who have either delayed or unsafe oral feeding skills, resulting in prolonged hospitalization and/or surgical placement of a gastric tube for administration of enteral nutrition. Despite the fact that the vast majority of infants in the NICU are at risk for these morbidities, research on neonatal feeding is relatively scarce compared to other complications of prematurity, such as bronchopulmonary dysplasia [5] and necrotizing enterocolitis [6], that almost exclusively affect a much smaller percentage of infants born at <32 weeks' gestation and/or at extremely low or very low birthweights [7].

Currently, newborns rely on the interpretation of subjective feeding cues by their caregivers to determine when it is safe to orally feed [8, 9]. Our understanding of the complexities of oral feeding maturation in the developing infant at the molecular level is largely unknown. Thus, there is an opportunity to incorporate emerging molecular techniques with conventional clinical approaches to improve our understanding of the complexities of oral feeding, and to develop objective diagnostic assays to accurately assess neonatal oral feeding skills. 

Previously, our laboratory described the enormous amount of real-time global developmental information available from premature infants through noninvasive salivary gene expression analyses [10]. Although in the initial study we did not specifically target gene transcripts involved in oral feeding, this original discovery-driven research identified key regulatory genes, as well as novel pathways, associated with oral feeding in the preterm infant. 

In the two years since this initial work was published, our understanding of the physiological functions of the genes initially identified continues to improve. Newly published reports on gene functions and their association with oral feeding have emerged. This has prompted a targeted reexamination of the data to identify previously unrecognized genes in neonatal saliva that may correlate to oral feeding success. Identifying these genes and their associated biological and physiological pathways may not only lead to objective, noninvasive salivary biomarkers that accurately predict oral feeding readiness, but may also highlight aberrant developmental pathways that correlate with pathological feeding behavior in the newborn. 

## 2. Materials and Methods

This study was approved by the Tufts Medical Center Institutional Review Board. Parental consent was obtained for all enrolled subjects (*n* = 5). Salivary samples were collected and processed as previously described [10]. Gestational age at birth of the subjects ranged from 28 to 32 weeks. There were two females and three males in this data set. Pertinent clinical information, including medical complications, of each subject can be found in Table 1. Salivary samples were collected from each subject from the following time points: (1) no feeds; (2) partial gastric feeds; (3) full gastric feeds; (4) some oral feeds; (5) advanced oral feeds. Salivary RNA was extracted, amplified, and hybridized onto the Affymetrix HG U133 2.0 Plus gene expression arrays. 

### 2.1. Analysis

All arrays (*n* = 25) underwent normalization and bioinformatic analysis as previously described [10]. Genes that were shown to statistically significantly alter their gene expression over time at a false discovery rate *P* value of <0.05 were identified (*n* = 9,286). There were 5,764 up-regulated and 3,522 down-regulated genes that met statistical criteria and altered their expression profile as the subjects matured through the feeding stages. For the purpose of this study, the up- and down-regulated gene lists were uploaded into the newest version of Ingenuity Pathway Analysis (IPA Content version: 11904312, release date 12-15-2011). IPA then performed a functional analysis of the gene lists with a right-tailed Fisher's exact test to calculate a *P* value determining the probability that each biological function assigned to that data set was due to chance alone. This analysis provided a comprehensive biological assessment of the gene-gene interactions, gene functions and gene regulation in our data sets.

In order to provide a targeted analysis of genes most likely to be related to oral feeding skills, genes that clustered into statistically significantly biological pathways by IPA were only considered if they met the following criteria: (a) they were in the five most statistically significantly up- or down-regulated physiological pathways; (b) they were associated with the key terms “feeding,” “digestion,” and/or “development” in the IPA analysis. Thus, genes had to statistically significantly alter their gene expression over time, and be identified by IPA as significantly clustering into pathways believed to be associated with oral feeding skills to be considered in this analysis. Genes that met these criteria were further reviewed by the author with the use of IPA, PubMed, and EntrezGene to better understand their functions and possible roles in neonatal oral feeding. 

## 3. Results and Discussion

From 2010 when the data was initially published until present, the functions of 4% of the genes in the original data sets have been modified. As such, those genes are no longer considered valid for previously identified biological functions, canonical pathways, or networks. This recategorization of genes is a direct result of emerging literature and an improved understanding of gene function, gene-gene interaction, and gene regulation. Although it is common for gene expression analyses to change over time, the slight difference in gene analysis from this data set has allowed for the identification of genes not previously known to be associated with neonatal oral feeding. The most significant physiologic pathways identified in this analysis highlight the complexity of oral feeding and involve not only tissue and nervous system development, but key pathways involved in feeding behavior. The five most statistically significantly up-regulated pathways were “behavior” (10^−10^ < *P* < 10^−2^), “nervous system development” (10^−9^ < *P* < 10^−2^), “tissue development” (10^−7^ < *P* < 10^−2^), “embryonic development” (10^−7^ < *P* < 10^−2^), and “organ development” (10^−7^ < *P* < 10^−2^). The five most statistically significantly down-regulated pathways were “hematological system development and function” (10^−10^ < *P* < 10^−3^), “hematopoiesis” (10^−10^ < *P* < 10^−3^), “lymphoid tissue structure and development” (10^−9^ < *P* < 10^−5^), “organismal survival” (10^−9^ < *P* < 10^−4^), and “cell-mediated immune response” (10^−8^ < *P* < 10^−5^). Each pathway is inclusive of related subcategories. For example, within nervous system development, the subcategories “development of cranial nerve” (*P* < 0.001) and “development of olfactory receptors” (*P* < 0.01) were found. There were 1,807 up-regulated genes that met the search criteria; 379 down-regulated genes were also considered.

One of the most novel aspects of both the current and previous analysis is the prominent role of “behavior” in neonatal oral feeding. Of all the statistically significantly upregulated pathways identified, genes associated with “behavior” were the most significant. Within this pathway, a subcategory entitled “feeding” was highlighted (*P* < 10^−5^). Genes within this pathway were associated with “hyperphagia,” “satiety,” “obesity,” and “weight gain” (Table 2). This novel pathway suggests that oral feeding in the newborn is neither merely reflexive nor solely dependent upon oral musculature and nervous system development. Rather, newborns rely, in part, on complex neurological signaling related to hunger, satiety, and energy expenditure for successful oral feeding. This makes biological sense. During the first year of life, a healthy term newborn will gain 200% or more of its birth weight. A preterm infant may gain upwards of 300% of his or her birth weight. The enormous amount of caloric intake required for such exponential growth is unique to the newborn period of the human lifespan. Thus, it is not surprising that the most statistically significant pathway in our analysis is related to biological mechanisms driving feeding behavior. 

In the five most significantly up-regulated physiological pathways, new gene functions have also emerged. For example, in the prior study, the development of only the trigeminal nerve (Cranial Nerve [CN] V) was highlighted. At the time, we speculated that the development of this nerve, which provides motor innervation to the muscles of mastication, was essential for proper coordination of the suck- and swallow-reflex. In this updated analysis, we not only see upregulation of genes involved in the development of the trigeminal nerve, but also have identified genes involved in the developing facial (CN VII) and glossopharyngeal (CN IX) nerves (Table 3). Each of these nerves is known to be essential for safe coordination of swallowing with respiration, with CN VII innervating the sensory component of the facial mask, and CN IX providing sensory taste fibers to the posterior tongue. In addition, the subcategory “development of cranial nerve” was new to this analysis. Here, genes associated with the oculomotor (CN III) and vestibulocochlear (CN VIII) nerves were identified. Sensory development was also prominent in this targeted reanalysis of the data. There is an upregulation of genes involved in the developing eye (*P* < 0.01) and ear (*P* < 0.02), as well as the olfactory system (*P* < 0.01). Genes involved in olfactory receptor development were significantly upregulated as infants matured and learned to orally feed. This, too, is of great interest as emerging literature supports the role of the infant's olfactory system in the establishment of successful breastfeeding [11]. 

Genes involved in the developing nervous, skeletal and muscle systems were also highlighted in this analysis. There was a wide range of nervous system functions including the development of the brain, spine, central nervous system, neurons, neurites, and ganglions. Development of striated muscle (*P* = 0.009) was significantly upregulated over time, as was developing bone (*P* = 0.006) and cartilage (*P* = 0.005). One of the limitations of this study is that neonatal subjects were followed over several weeks while in the NICU. During that time, there were global developmental processes occurring as the infants matured. However, by limiting salivary collection to five predefined feeding stages, there was an opportunity to capture, in real-time, gene expression changes directly related to the attainment of safe oral feeding skills. These data support the complexity of oral feeding and further demonstrate how multiple processes involved in oral feeding may be monitored simultaneously in the newborn at the molecular level.

Of note, statistically significant down-regulated genes were related only to the developing hematological and lymphoid system. There was no obvious link between lymphoid and/or hematological development and oral feeding success that appeared when reviewing the down-regulated pathways and their associated subcategories. While it is possible that the development of the gastrointestinal immune system plays an important role in readiness to feed, our current understanding of these genes prohibits further speculation on their role, if any, in neonatal oral feeding skills. 

This research demonstrates that with the advent of recent technical advances and high-throughput screening methods, we are able to monitor, in real-time, normal and aberrant developmental processes occurring in the newborn from mere drops of saliva. Utilizing this information for the development of noninvasive salivary diagnostic panels is a novel and exciting aspect of translational medicine. Indeed, salivary diagnostic platforms are currently in development for adult patients with oral cancer, breast cancer, Sjögren's disease, pancreatic cancer, melanoma, nonsmall cell lung cancer, acute myocardial infarction, diabetes, and ovarian cancer [12–17]. These platforms are designed to be utilized at the bedside in order to give an accurate diagnosis within minutes with the use of point-of-care technology currently in development [18]. While these platforms are clinically important, none targets a neonatal or even a pediatric patient population. Yet neonates, with their limited blood volumes and clinical fragility, are the ideal patient population on which to apply this technology.

**Figure 1:**
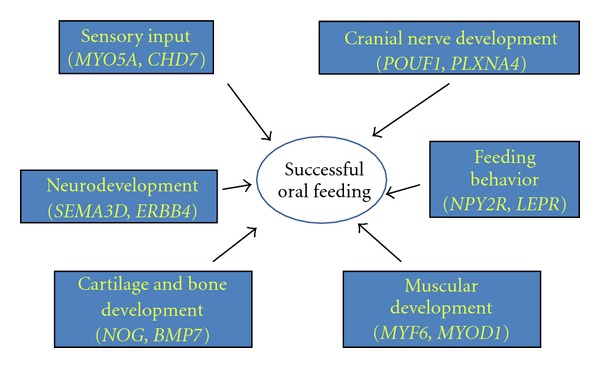
Through salivary gene expression analyses, genes involved in multiple developmental systems that are required for successful neonatal oral feeding can be monitored noninvasively and simultaneously. Combining gene targets, such as those identified, may ultimately lead to a noninvasive, objective, and accurate salivary diagnostic platform to determine readiness to orally feed in the newborn.

Compared to our prior analysis which examined global developmental processes in the newborn, the present study focused solely on genes believed to be involved in the attainment of successful oral feeding skills. This targeted analysis of the data is laying the foundation for the development of a neonatal oral readiness to feed salivary diagnostic platform. For example, our laboratory recently determined that salivary detection of one of the genes identified in this data set, neuropeptide Y2 receptor (*NPY2R*), a known hypothalamic regulator of feeding behavior, has a 95% positive predictive value for immature oral feeding skills [19]. A limitation to this biomarker is that if undetected in saliva, it is only 27% accurate in determining a safe and effective oral feeding pattern. However, as this work moves forward, other genes identified in this targeted analysis could be incorporated into a comprehensive platform, not only for the development of an accurate and objective diagnostic assay, but also to improve our understanding of aberrant feeding patterns in the newborn (Figure 1).

## 4. Conclusion

Neonatal salivary transcriptomic analysis provides noninvasive and objective information about the learning process of oral feeding in the newborn at the molecular level. These data further confirm the complexities of oral feeding and suggest that the development of feeding behavior is a novel and essential biological component to successful oral feeding. This research lays the foundation for the development of an objective, noninvasive assay for the determination of readiness to feed in the neonatal population in order to reduce morbidities and improve care and outcomes.

## Figures and Tables

**Table 1:** Pertinent clinical information of subjects.

Subject	Gender	Gestational age (weeks)	Birthweight (g)	Medical complications
1	Male	29	1389	Respiratory distress syndrome (RDS), apnea, anemia, and hyperbilirubinemia
2	Female	28 3/7	942	RDS, hyperbilirubinemia, anemia, and apnea
3	Male	28 3/7	1123	RDS, hyperbilirubinemia, apnea, anemia, and retinopathy of prematurity (stage 2, zone 2)
4	Female	32	1683	RDS, hyperbilirubinemia, apnea, and anemia
5	Female	32	1379	RDS, hyperbilirubinemia, apnea, and anemia

**Table 2:** Genes involved in feeding behavior (*P* < 10^−5^).

Gene	Gene symbol	Relevant biological function
Angiotensin I converting enzyme (peptidyl-dipeptidase A) 1	ACE	This gene encodes an enzyme involved in catalyzing the conversion of angiotensin I into a physiologically active peptide angiotensin II.
Cholecystokinin A receptor	CCKAR	In the central and peripheral nervous system this receptor regulates satiety.
Cannabinoid receptor 1 (brain)	CNR1	Animal experiments utilizing receptor antagonists resulted in suppressed food and water intake with concurrent decreased body weight.
Corticotropin releasing-hormone	CRH	Corticotropin-releasing hormone is secreted by the paraventricular nucleus (PVN) of the hypothalamus in response to stress.
Corticotropin releasing-hormone receptor 1	CRHR1	The encoded protein is essential for the activation of signal transduction pathways that regulate diverse physiological processes including obesity.
Diencephalon/mesencephalon homeobox 1	DMBX1	This gene is known to be involved in adult feeding behavior and may play a role in brain and sensory organ development.
Free fatty acid receptor 1	FFAR1	The encoded protein is a receptor for medium and long chain free fatty acids and may be involved in the metabolic regulation of insulin secretion.
Glutamate decarboxylase 2 (pancreatic islets and brain, 65 kDa)	GAD2	This gene has been shown to be a candidate gene for obesity in humans.
Galanin-like peptide	GALP	This gene is involved in biological processes including hypothalamic regulation of metabolism.
Galanin receptor 3	GALR3	The neuropeptide galanin modulates a variety of physiologic processes including feeding behavior.
Glucagon	GCG	Glucagon is a pancreatic hormone that counteracts the glucose-lowering action of insulin by stimulating glycogenolysis and gluconeogenesis.
Growth hormone secretagogue receptor	GHSR	The encoded protein may play a role in energy homeostasis and regulation of body weight.
Glucagon-like peptide 1 receptor	GLP1R	This gene is involved in energy reserve metabolic processes and feeding behavior.
Glutamate receptor, ionotropic, N-methyl D-aspartate 2B	GRIN2B	NMDA receptor channel is involved in the activity-dependent increase in the efficiency of synaptic transmission thought to underlie certain kinds of memory and learning.
Hypocretin (orexin) receptor 2	HCRTR2	The protein encoded by this gene is a G protein coupled receptor involved in the regulation of feeding behavior.
Histamine receptor H3	HRH3	This gene encodes an integral membrane protein and can regulate neurotransmitter release.
5-hydroxytryptamine (serotonin) receptor 1A, G protein-coupled	HTR1A	Gene has been shown to be involved in control of food intake in obese rats.
5-hydroxytryptamine (serotonin) receptor 2C, G protein-coupled	HTR2C	This gene is involved in feeding behavior.
Interleukin 1 receptor antagonist	IL1RN	The protein encoded by this gene is a member of the interleukin 1 cytokine family.
Janus kinase 1	JAK1	Knockout mice of this gene exhibit decreased nursing behavior.
Junctophilin 1	JPH1	This gene is involved in muscle organ development.
Lactalbumin, alpha	LALBA	This gene encodes alpha-lactalbumin, a principal protein of milk.
Leptin receptor	LEPR	This protein is a receptor for leptin and is involved in the regulation of fat metabolism.
Melanin-concentrating hormone receptor 1	MCHR1	The gene is involved in the neuronal regulation of food consumption.
NK2 homeobox 1	NKX2-1	This gene is involved in brain development and feeding behavior.
Neuropeptide Y receptor Y1	NPY1R	Neuropeptide Y exhibits a diverse range of important physiologic activities including regulation of food consumption.
Neuropeptide Y receptor Y2	NPY2R	This gene is involved in regulating feeding behavior.
Neurotrophic tyrosine kinase, receptor, type 2	NTRK2	This gene is involved in feeding behavior. Mutations in this gene have been associated with obesity.
Opioid receptor, kappa 1	OPRK1	This gene is involved in regulating behavior.
Peroxisomal biogenesis factor 13	PEX13	This gene is involved in suckling behavior.
POU class 4 homeobox 1	POU4F1	This gene is highly expressed in the developing sensory nervous system.
Prolactin releasing hormone	PRLH	This gene is involved in feeding behavior and regulates multicellular organism growth.
Prostaglandin E receptor 3 (subtype EP3)	PTGER3	This receptor may have many biological functions, which involve digestion and the nervous system.
PTK2 protein tyrosine kinase 2	PTK2	This gene plays a role in glucose response, fat-cell differentiation, and the growth hormone receptor signaling pathway.
Peptide YY	PYY	This gene is involved in digestion and feeding behavior.
Solute carrier family 18 (vesicular monoamine), member 2	SLC18A2	This gene is involved in glucose homeostasis and response to starvation.
Solute carrier family 27 (fatty acid transporter), member 5	SLC27A5	This gene is involved in digestion.
Tachykinin receptor 1	TACR1	This gene is involved in eating behavior.
Tyrosine hydroxylase	TH	This gene plays a role in eating behavior.
Thyrotropin-releasing hormone	TRH	This gene plays a role in eating behavior.
Transient receptor potential cation channel, subfamily M, member 5	TRPM5	This gene plays an important role in taste transduction.

**Table 3:** Cranial nerve development and morphogenesis (10^−3^ < *P* < 10^−2^).

Gene	Symbol	Relative biological functions
Glossopharyngeal morphogenesis and development (10^−3^ < *P* < 10^−2^)		

Sema domain, immunoglobulin domain (Ig), short basic domain, secreted, (semaphorin) 3D	SEMA3D	Nervous system development
Plexin A4	PLXNA4	Cranial nerve morphogenesis; facial nerve morphogenesis (CN VII); glossopharyngeal nerve (CN IX) morphogenesis
Homeobox D3	HOXD3	Glossopharyngeal nerve (CN IX) morphogenesis
Homeobox B3	HOXB3	Glossopharyngeal nerve (CN IX) morphogenesis

Survival and development of trigeminal nerve and ganglion (10^−3^ < *P* < 10^−2^)		

B-cell CLL/lymphoma 2	BCL2	Neurodegeneration
Glial cell derived neurotrophic factor	GDNF	Axon guidance; neuron differentiation; neuron projection development
Neurturin	NRTN	Neuron projection development; axon guidance
POU class 4 homeobox 1	POU4F1	Neuron differentiation
POU class 4 homeobox 2	POU4F2	Trigeminal nerve development (CN V); suckling behavior
Solute carrier family 6 (neurotransmitter transporter, creatine), member 8	SLC6A8	Neurotransmitter transport
Noggin	NOG	Axon guidance; face morphogenesis; regulation of neuron differentiation

Cranial nerve development and morphogenesis ( *P* < 10^−3^)		

GLI family zinc finger 3	GLI3	Optic nerve morphogenesis (CN II); palate development; tongue development
Cholinergic receptor, nicotinic, beta 2 (neuronal)	CHRNB2	Conditioned taste aversion; optic nerve morphogenesis; vestibulocochlear nerve development (CN VIII)
Chromodomain helicase DNA binding protein 7	CHD7	Face development; nose development; palate development; sensory perception of sound; development in camera-type eye
Hairy and enhancer of split 1, (Drosophila)	HES1	Oculomotor nerve development (CN III); pharyngeal system development; auditory receptor cell differentiation and determination; cochlea development
Neuropilin 2	NRP2	Semaphorin-plexin signaling pathway
Sema domain, immunoglobulin domain (Ig), short basic domain, secreted, (semaphorin) 3D	SEMA3D	Nervous system development
Homeobox B3	HOXB3	Glossopharyngeal nerve (CN IX) morphogenesis
Homeobox D3	HOXD3	Glossopharyngeal nerve (CN IX) morphogenesis
Plexin A4	PLXNA4	Cranial nerve morphogenesis; facial nerve morphogenesis (CN VII); glossopharyngeal nerve morphogenesis (CN IX)
Thyroid hormone receptor, beta	THRB	Sensorineural hearing loss
v-erb-a erythroblastic leukemia viral oncogene homolog 4 (avian)	ERBB4	Central nervous system morphogenesis and olfactory bulb interneuron differentiation
Homeobox D3	HOXD3	Glossopharyngeal nerve morphogenesis (CN IX)
POU class 4 homeobox 1	POU4F1	Neuron differentiation
Myosin VA (heavy chain 12, myoxin)	MYO5A	Visual perception
Sal-like 1 (Drosophila)	SALLI	Olfactory bulb development and interneuron differentiation; outer ear morphogenesis
